# Protein Kinase C ε Expression in Platelets from Patients with Acute Myocardial Infarction

**DOI:** 10.1371/journal.pone.0046409

**Published:** 2012-10-05

**Authors:** Cecilia Carubbi, Prisco Mirandola, Maria Mattioli, Daniela Galli, Nicola Marziliano, Piera Angelica Merlini, Daniela Lina, Francesca Notarangelo, Maria Rita Cozzi, Marco Gesi, Diego Ardissino, Luigi De Marco, Marco Vitale, Giuliana Gobbi

**Affiliations:** 1 Department of Biomedical, Biotechnological and Translational Sciences, University of Parma, Parma, Italy; 2 Division of Cardiology, Ospedale Niguarda, Milano, Italy; 3 Division of Cardiology, Azienda Ospedaliero-Universitaria di Parma, Parma, Italy; 4 Department of Laboratory Medicine, CRO National Cancer Institute, Aviano, Italy; 5 Department of Human Morphology and Applied Biology, University of Pisa, Pisa, Italy; Stem Cell Research Institute, Belgium

## Abstract

**Objective:**

Platelets play crucial roles in the pathophysiology of thrombosis and myocardial infarction. Protein kinase C ε (PKCε) is virtually absent in human platelets and its expression is precisely regulated during human megakaryocytic differentiation. On the basis of what is known on the role of platelet PKCε in other species, we hypothesized that platelets from myocardial infarction patients might ectopically express PKCε with a pathophysiological role in the disease.

**Methods and Results:**

We therefore studied platelet PKCε expression from 24 patients with myocardial infarction, 24 patients with stable coronary artery disease and 24 healthy subjects. Indeed, platelets from myocardial infarction patients expressed PKCε with a significant frequency as compared to both stable coronary artery disease and healthy subjects. PKCε returned negative during patient follow-up. The forced expression of PKCε in normal donor platelets significantly increased their response to adenosine diphosphate-induced activation and adhesion to subendothelial collagen.

**Conclusions:**

Our data suggest that platelet generations produced before the acute event retain PKCε-mRNA that is not down-regulated during terminal megakaryocyte differentiation. Results are discussed in the perspective of peri-infarctual megakaryocytopoiesis as a critical component of myocardial infarction pathophysiology.

## Introduction

Myocardial infarction (MI) is an atherothrombotic disease determined by the interplay between an individual's genetic background, lifestyle and environment. Atherothrombosis, in turn, is the result of a complex pathological process that is characterized by endothelial dysfunction, atherosclerosis, and finally thrombus formation as the key event of acute MI. Monocytes and platelets are the principal cells involved in these events. Platelets, produced by the cytoplasmic fragmentation of bone marrow megakaryocytes (MK), are essential for primary hemostasis, to repair microvascular damages and to initiate physiological thrombus formation. Calcium mobilization is required for stable platelet incorporation into the developing thrombus. Platelets therefore play a pivotal role in the thrombus formation, as well as in the plaque development [1] from the very beginning of atherosclerotic disease. A variety of platelet functions have been associated with PKC activity [2]. PKC activity, in synergy with Ca^2+^, regulates the secretion of dense and α-granules following platelet stimulation with phospholipase C-stimulating agonists, like collagen and thrombin [3]–[5]. Secretion of ADP, fibrinogen, and other stored compounds, in turn, enhance the activation process [6], [7]. PKC-mediated protein phosphorylation also induces the conformational changes of integrin αIIbβ3 required for fibrinogen binding and platelet aggregation [8], [9]. Activated integrins, in turn, stimulate PKC via outside-in signaling, resulting in filopodial formation and platelet spreading [10], [11]. Ca^2+^-dependent PKC isoforms contribute to platelet aggregation at least in two different ways, directly via integrin phosphorylation and indirectly via granule secretion. It has been reported that PKC is involved in Ca^2+^ flux in platelets [3], [12], while under flow conditions PKC contributes to the stable adhesion of platelets to collagen but not to their initial attachment to the vessel wall [13]. Finally, it has been demonstrated that platelet PKCs have a dual controlling role in thrombus formation, balancing the proaggregatory and procoagulant properties of thrombi [14] suggesting that the different PKC isoforms present in platelets participate to distinct activatory or suppressive pathways, the latter of which are mediated by one or more non-classical PKC isoforms [14]. Notwithstanding these research efforts, a clear picture of the role of the different PKC isoforms in platelets is still lacking. Human platelets in normal conditions predominantly express four PKC isoforms, namely α, β, δ, θ [15], [16], which phosphorylate multiple proteins during platelet activation [17], [18]. PKCδ is phosphorylated in response to GPVI and PAR receptors, but not αIIbβ3 activation [19]–[21]. Differently, PKCθ is phosphorylated in response to collagen, the snake toxin Alboaggregin A (which interacts with both GPVI and GP-Ib-IX-V) and αIIbβ3 [22], [23]. The expression of PKCε in human platelets is still a matter of debate, as most Authors do not find it [11], [24], while others reported its presence [22], [25]. Among novel PKCs, the expression and function of the epsilon isoform are not thoroughly understood. Although it is now well established that mouse platelets express PKCε [24], functional data are still contradictory. In platelets from PKCε null mouse, Pears et al showed a marked inhibition of aggregation and dense granule secretion in response to GPVI agonists but no significant functional change in response to ADP [24]. At the opposite, recently Bynagari-Settipalli et al [26] showed an increase in ADP-induced aggregation and secretion in platelets from PKCε null mice. Indeed, signaling through GPVI suggests a role for PKCε in the initial steps of thrombus formation in mouse platelets. Of note, in human monocytes the adhesion to endothelial cells involves PKCε signaling [27].

Although enucleated, platelets retain cytoplasmic mRNA, accounting for more than 2000 transcripts [28], and maintain functionally intact protein translation capabilities, including an abundant variety of microRNA [29]. On this basis, the emerging concept is that platelet protein synthesis might be relevant in the pathophysiology of acute cardiovascular events [30], [31]. Although it has been demonstrated that platelets may retain small pre-mRNA, like Tissue Factor-1 and IL-1β pre-mRNA that can be spliced following in vitro platelet activation [32], [33], most platelet protein synthesis refers to recent megakaryocytopoiesis.

Therefore, on the basis of : i) the idea that in the progression of the cellular and molecular events that characterize acute MI, the platelets generated around the acute event might be characterized by a specific profile of gene expression; ii) our previous works on PKCε expression during MK differentiation [34], [35] and erythroid differentiation [36], [37]; iii) the functional data on platelet PKCε available in the mouse, we hypothesized that an ectopic expression of PKCε might be present in platelets from MI patients.

## Methods

### Patients

Three groups of subjects were studied: 1) twenty-four acute myocardial infarction patients (MI) with an ST-segment elevation; 2) twenty-four patients with newly-diagnosed stable CAD (sCAD) and 3) twenty-four healthy subjects (HD). Patients were enrolled at the Cardiology Division of the Azienda Ospedaliero-Universitaria of Parma after written informed consent was obtained and the study was performed according to the Declaration of Helsinski. The protocol was approved by the unique Local Ethical Committee of the Ospedale Maggiore of Parma and University of Parma. Blood collection from MI patients was accomplished within 12 hours from the acute event (in most cases within 3–4 hours) and before any invasive procedure or pharmacological treatment was performed. Previous antiplatelet therapy was an exclusion criteria in all groups. Patients characteristics and their cardiovascular risk factors are reported in **Table 1**.

**Table 1:** Demographic characteristics and cardiovascular risk factors.

Cardiovascular risk factors	MI	sCAD	HD
	N = 24 (%)	N = 24 (%)	N = 24 (%)
**Mean age**	68.38	67.66	67.38
Median (50°percentile)	68.0	68.5	69.5
**Gender**			
-Male	18 (75.00)	16 (66.67)	16 (66.67)
-Female	6 (25.00)	8 (33.33)	8 (33.33)
**Family history of cardiovascular disease**			
-Yes	7 (29.17)	11 (45.84)	4 (16.67)
-No	17 (70.83)	13 (54.16)	20 (83.33)
**Diabetes**			
-Yes	3 (12.50)	5 (20.83)	1 (4.16)
-No	21 (87.50)	19 (79.17)	23 (95.84)
**Smoking**			
-Yes	20 (83.33)	15 (62.50)	11 (45.83)
-No	4 (16.66)	9 (37.50)	13 (54.17)
**Hypertension**			
-Yes	13 (54.16)	19 (79.17)	14 (58.33)
-No	11 (45.83)	5 (20.83)	10 (41.66)
**Body mass index**			
-normal	9 (37.50)	10 (41.66)	10 (41.66)
-pre-obese	14 (58.33)	12 (50.00)	12(50.00)
-obese	1 (4.16)	2 (8.33)	2 (8.33)
**Hypercholesterolemia**			
-Yes	8 (33.33)	17 (70.84)	5 (20.83)
-No	16 (66.66)	7 (29.16)	19 (79.16)

Fifty ml of citrate anti-coagulated blood samples were taken from patients and controls (collected in Vacutainers, 3.8% sodium citrate final concentration; BD Vacutainer, Becton Dickinson, San Diego, CA) for subsequent analyses.

### Platelet activation

Aliquots of whole blood samples were stained with anti-CD62p monoclonal antibody (mAb) as a marker of platelet activation and α-granule release and analyzed by flow cytometry [38]. Briefly, 1∶100 PBS-diluted whole blood was incubated with the mAb CD62p-FITC (anti P-selectin, Pharmingen Becton Dickinson, San Diego, CA) in the presence of incremental doses of ADP (0; 1,25; 2,5; 5 µM). After 20 min at room temperature, 400 µl of 2% buffered paraformaldehyde was added for fixation.

Analysis of the samples was performed by an Epics XL flow cytometer (Beckman Coulter, Fullerton, CA) and the Expo ADC software (Beckman Coulter). In some experiments, the absolute number of surface antigens expressed/cell was calculated. To this purpose, the flow cytometer was calibrated with a set of standardized beads (DAKO, Glostrup, Denmark) each with a known amount of fluorochrome (either FITC or PE) expressed in units of MESF (Molecules of Equivalent Soluble Fluorescein). Thus, a standard curve was constructed by plotting MESF values for the beads against the median channel in which the peak was displayed.

### Platelet purification

All the remaining blood samples were centrifuged at 160 g for 20 minutes at room temperature (RT), to obtain platelet rich plasma (PRP). Platelets were then purified by negative separation using magnetic beads coated with anti-CD45 antibodies (Dynabeads®, Invitrogen, Carlsbad, CA), to deplete nucleated cells. Briefly, PRP were stained with the magnetic beads-coated mAb anti-CD45 for 20 min at RT on a rotator. Purified platelets were washed 3 times in PBS/BSA solution, counted, tested for purity by anti-CD41 staining and flow cytometry analysis (only samples where CD41^+^ cells >98% were used), and finally processed for RNA extraction.

### RNA isolation

Higly purified platelets were treated with an appropriate amount of TRIzol™ (Invitrogen) for cell lysis and RNA extraction, following the manufacturer's protocol. Briefly, chloroform was added to TRIzol™-treated samples and centrifuged at 12.000 g for 15 min at 4°C. The acqueous phase, containing RNA, was transferred in a new tube and added with an equal volume of isopropanol. After incubation, the samples were centrifuged at 12.000 g for 15 min at 4°C, to obtain RNA pellets that were washed and resumed in DEPC-treated water for quantification by spectrophotometer.

### Amplification of RNA for PKCε gene expression analysis

The isolated RNA was both positively and negatively tested for cell population purity by Reverse-Transcription PCR (RT-PCR). A standard set of primers was used to test platelet RNA (amplification of CD41) or contaminant cells RNA (amplification of CD45 for nucleated cells).

Platelet RNA purification was followed by reverse transcription and RT-PCR to yield complementary DNA (cDNA). From the cDNA sample, cRNA was synthesized by in vitro transcription (IVT), and then analyzed for PKCε gene expression. Briefly, 1 µg total RNA was reverse transcribed with MMV reverse transcriptase and subjected to PCR amplification to detect CD41, CD45 and PKCε cDNA.

PCR were performed under the following reaction conditions: 95°C for 30 sec, 56°C for 30 sec, 72°C for 30 sec and a final extension at 72°C for 5 min. We used 35 cycles of amplification. The sequences of primers used for PCR were: **CD41**: 5′-GCAAT GTCGA GGGCT TTGAG-3′ (sense) and 5′-GGCTG TTCTT GCTCC GTATC-3′ (antisense); **CD45**: 5′-GGAAG TGCTG CAATG TGTCA TT-3′ (sense) and 5′-CTTGA CATGC ATACT ATTAT CTGAT GTCA-3′ (antisense); **PKCε**: 5′-CAATG GCCTT CTTAA GATCA AAA-3′ (sense) and 5′-CCTGA GAGATC GATGATC ACATAC-3′ (antisense). Primers used for PKCε RT-PCR amplify exon 1 and exon 2 sequences flanking the first intronic sequence of the gene, which is 190,000 bp.

### Quantitative analysis for PKCε gene expression by Real-Time PCR

Equal quantities of RNA for each sample were retro-transcribed with Malone Murine Leukemia Virus Reverse Transcriptase (Promega, Madison WI, USA) according to manufacturer'instructions. Two µl of 1∶1 cDNA dilution were used to perform real-time PCR with GoTaq® qPCR master mix (Promega) and 200 nM of each primer in Applied Biosystems StepOne real-time machine (Applied Biosystems, Carlsbad, CA). Each reaction was performed in triplicate and mean Ct values were considered for quantitation. Relative gene expression was analysed using comparative Ct experiment software subset following manufacturer's instructions.

The sequences of primers used for PCR were: **CD41**: 5′-GCAAT GTCGA GGGCT TTGAG-3′ (sense) and 5′-GGCTG TTCTT GCTCC GTATC-3′ (antisense); **CD45**: 5′-GGAAG TGCTG CAATG TGTCA TT-3′ (sense) and 5′-CTTGA CATGC ATACT ATTAT CTGAT GTCA-3′ (antisense); **PKCε**: 5′-CACCA TCCAG TTTGA GGAGC-3′ (sense) and 5′-CGACC CTGAG AGATC GATGA -3′ (antisense).

### cDNA PKCε sequencing

The coding *PKCε* sequence (*ENST00000306156*; cDNA 11->397) was amplified from the cDNA as described above and the amplicons underwent direct sequencing analysis performed using the BigDye Terminator Cycle sequencing kit V 3.1 (Applied Biosystems) on a 3130*Xl* Genetic Analyzer (Applied Biosystems), following the manufacturer's directions. Electropherograms were analysed using the SeqScape Software (Applied Biosystems) and sequences were blasted using the CLUSTAL W algorithm (www.ebi.ac.uk/clustalw).

### Protein extraction and western blot

Total proteins were extracted from 1 ml (450×10^3^/µl) of purified platelets. Briefly, 1 ml of isolated platelets from each sample was collected and centrifuged at 1700 rpm for 10 min. The pellets were then suspended in a cell lysis buffer (50 mM Tris-HCl, pH 7.4; 1% NP-40; 0.25% sodium deoxycholate; 150 mM NaCl; 1 mM EDTA; 1 mM PMSF; 1 mM Na_3_VO_4_; 1 mM NaF) supplemented with fresh protease inhibitors and protein concentration was determined using BCA™ protein assay kit (Pierce, Rockford, IL). Fifty µg of proteins from each sample were then migrated in 5% SDS-acrylamide gels and blotted onto nitrocellulose filters.

Blotted filters were blocked and incubated with specific primary antibodies diluted as described in maunfacturers' protocols. Specifically, rabbit polyclonal anti-PKCε (Upstate, Lake Placid, NY) and mAb anti-β-actin (Sigma, Saint Luis, MO) were diluted 1∶5000. Filters were washed and further incubated for 1.5 hours at room temperature with 1∶5000 peroxidase-conjugated anti-rabbit or with 1∶2000 peroxidase-conjugated anti-mouse IgG (Pierce) in the primary antibody working solution at RT. Specific reactions were revealed with the ECL Supersignal West Pico Chemiluminescent Substrate detection system (Pierce).

### PKCε protein expression in platelets by flow cytometry

Aliquots of PRP samples were stained with: i) anti-CD41a monoclonal antibody (mAb), as a marker of platelet population; ii) Thyazole Orange (TO), to identify reticulated platelets; iii) rabbit anti-PKCε monoclonal antibody, to test the presence of PKCε protein; and analyzed by flow cytometry. Briefly, 200 µl of PRP were washed and treated with IntraPrep™ Permeabilization Reagent (Immunotech, Marseille, France), following manufacturer's protocol. One-hundred µl of Reagent 1 (fixation reagent) were added to the samples. After 15 min of incubation at room temperature, samples were washed and incubated with 100 µl of Reagent 2 (permeabilization reagent), 10 µl CD41a-Cy5 (Becton Dickinson, San Diego, CA) and 1 µl of affinity purified rabbit anti-PKCε Ab (Novus Biologicals, Littelton, CO); negative control was incubated with 100 µl of Reagent 2, 10 µl isotype-matched IgG-Cy5 (Immunotech) and 1 µl of an isotype-matched Ab (rabbit IgG, Sigma) for 45 min at room temperature in the dark. After a washing step, 1 µl of affinity purified goat anti-rabbit-PE Ab (Beckman Coulter) was added and the samples were incubated for 45 min at room temperature in the dark. Finally, the samples were washed and a solution10 ng/ml of TO (Sigma) was added, in the presence or absence of 10 mg/ml RNAsi (negative control), and incubated for 30 min at room temperature in the dark and analysed by Flow Cytometry. Working dilutions of all reagents were previously determined with serial dilution tests. All samples were analysed on an FC500 flow cytometer (Beckman Coulter).

### Platelet transfection with PKCε protein

Human recombinant PKCε (rhPKCε; GenWay Biotech, San Diego, CA) was transfected into purified platelets using Proteojuice protein transfection reagent (Novagen, Podenzano, Italy), according to manufacturer's protocols. For each transfection, 1 ml of PRP was centrifuged at 1800 rpm for 7 min, the supernatant was removed and pellets were washed with serum-free medium. Subsequently, samples were centrifuged at 1800 rpm for 7 min and medium was completely removed. The transfection mixture was prepared as follows: 25 µl of serum-free medium were added to 1 µg of PKCε protein (or nothing, as negative control) and 1,25 µl of ProteoJuice protein transfection reagent. After 20 min of incubation at room temperature, 225 µl of serum-free medium were added to the transfection mixture. Finally, the platelet samples were incubated with the mix at 37°C, 5% CO_2_ for 3,5 hours. After transfection, the samples were washed twice with serum-free medium to remove excess protein.

In some experiments, transfected and control platelets were re-added to platelet-deprived whole blood and aliquots were then used for platelet activation analysis by flow cytometry, as described above.

### Platelet adhesion analysis in shear rate system

Isolated PRP was treated for rhPKCε transfection, as described above. The procedures to prepare a washed erythrocyte suspensions have been described previously in detail [39]. PRP containing 2–8×10^8^ loaded platelets/ml was mixed with washed erythrocytes, to obtain a suspension with hematocrit of 42–45%, and apyrase (grade III; 142 ATPase U/mg of protein; Sigma) was added at the final concentration of 5 ATPase U/ml. The mixture was centrifuged at 1000 *g* for 15 min, the supernatant was discarded and the cell pellet was suspended in plasma. Suspensions of acid-insoluble fibrillar type I collagen from bovine achille tendon (Sigma) in 0.5M acetic acid (pH 2.8) were prepared as previously described [40] and two hundred microliters was used to coat glass coverslips for 60 minutes at 22–25°C in a humidified box. After coating, coverslips were washed with PBS and kept in a moist environment until assembled in a modified Hele-Shaw flow chamber [41]. The flow chamber was positioned on the stage of an inverted microscope equipped with epifluorescent illumination (Diaphot-TMD; Nikon Instech, Shinagawa-ku, Japan), an intensified CCD videocamera (C-2400-87; Hamamatsu Photonics, Shizuoka, Japan), and appropriate filters. The total area of an optical field corresponded to approximately 0.007 mm^2^. Blood cells were aspirated through the chamber with a syringe pump (Harvard Apparatus, Hollistone, MA) at a flow rate calculated to obtain the desired wall shear rate at the inlet. Platelet adhesion was measured using blood containing 10 µM mepacrine to render platelets fluorescent.

Three negative controls were run for each sample: i) untreated platelets; ii) platelets treated with a PKCε traslocation inhibitor peptide (Merck KgaA, Darmstadt, Germany) added to the trasfection mixture (1 ug of inhibitor peptide) with or, iii) without rhPKCε. Experiments were recorded in real time on videotape at the rate of 25 frames/s, which resulted in a time resolution of 0.04 s. Selected video sequences were also digitized in real time using a Matrox-Digisuite board (Matrox Graphics Inc., Dorval, Quebec, Canada).

Single frame images were captured from videotapes after an initial blood perfusion for 3 minutes on the different substrates. A threshold was applied to distinguish platelets from the background and the area occupied by all platelets in an image was measured using MicroImage (image-processing software; Tesi-Imaging srl, Venice, Italy).

### Statistical analysis

The variables were compared between the three groups of patients using One Way Anova and Bonferroni t-test for multiple comparisons. Where indicated the variables were compared using Mann-Whitney test. T-test for independent or correlated samples was used to compare some indicated data. All the results are expressed as means plus or minus SD. Chi-square analysis of contingency tables were used for PKCε mRNA expression analysis of frequency comparison in the three groups. All the statistical tests were performed at the 0.05 p-value.

## Results

### MI patients carry hyper-responsive platelets

**Figure 1:**
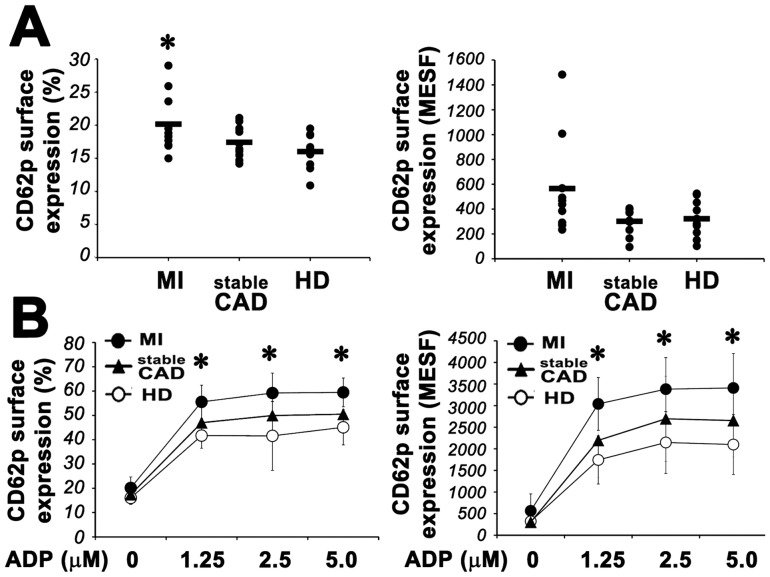
Platelet activation in MI patients. **Panel A:** **Left.** Flow cytometric analysis of platelet CD62p surface expression in patients with MI, sCAD and healthy donors. Cells were stained with specific mAb anti P-selectin, as described in Materials and Methods. Ten patients were analyzed for each group. **Right.** Quantification of CD62p expression on the surface of platelets from MI, sCAD and HD. Absolute numbers of surface antigens expressed/cell (MESF). (Anova and Bonferroni t-test; * P<0.05 MI *vs* HD; no significant differences were found between stableCAD and HD). **Panel B:** Platelet surface expression of CD62p in MI (•), sCAD (▴) and HD (○) patients in the presence of increasing doses of ADP. **Left** panel shows the percentage of positive cells. **Right** panel shows the absolute numbers of surface antigens expressed/cell (MESF). Data from 7 patients of each group are expressed as means ± S.D. (Anova and Bonferroni t-test; * P<0.05 MI *vs* HD; no significant differences were found between stable CAD and HD).

It is well known that patients with acute coronary syndromes carry hyper-responsive platelets [42], [43] and show systemic platelet activation [43]–[47]. We therefore first studied the activation state of platelets from a randomly selected sub-group of our patients and their sensitivity to agonistic stimuli. In agreement with the current literature, we found that platelets from MI patients express significantly higher surface levels of p-selectin than those from sCAD patients and healthy donors (**Fig. 1A**). Moreover, in MI patients CD62p expression was induced at significantly higher levels by sub-optimal doses of ADP (**Fig. 1B**).

### Platelets from MI patients contain mRNA for PKCε and express PKCε

**Figure 2:**
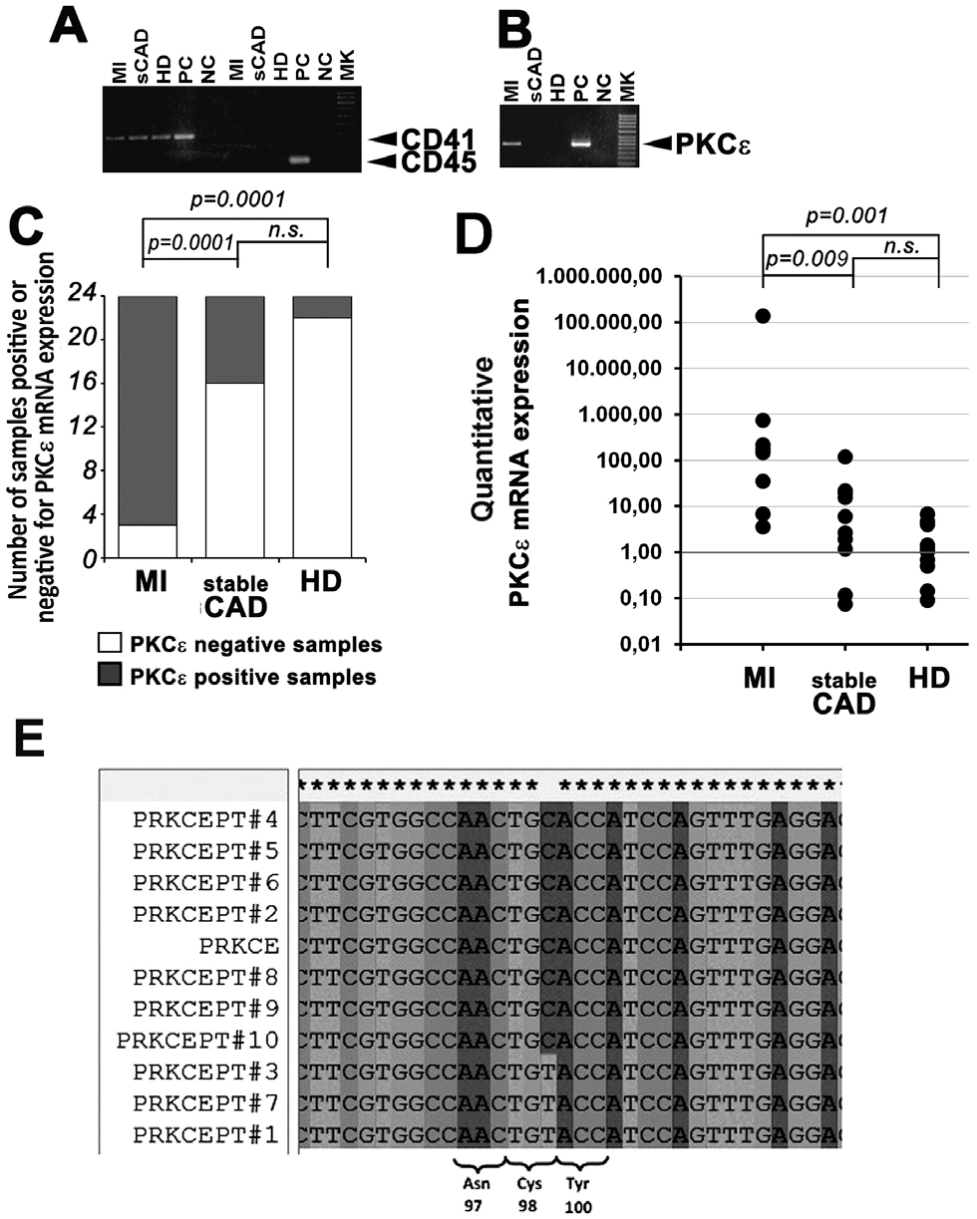
Platelets from MI patients contain mRNA for PKCε. **Panel A:** Representative RT-PCR analysis of CD41 and CD45 expression in MI, sCAD and healthy donor platelets. Equal amounts of total cDNA were amplified by PCR to detect the indicated mRNA expression. CD45 is not expressed in isolated platelets, indicating the absence of nucleated cell contaminants. **Panel B:** Representative RT-PCR analysis of PKCε expression in MI, sCAD and healthy donor platelets. Equal amounts of total cDNA were amplified by PCR to detect PKCε mRNA. Platelets from MI patients express PKCε. PC: positive control; NC: negative control. **Panel C:** Analysis of PKCε RNA expression in MI, sCAD and HD platelets. The samples positive to PKCε mRNA expression in MI, sCAD and HD were respectively 21, 8, 3 out of 72 (24 each group) (Chi-square test: p = 0.0001 MI *vs* HD; p = 0.0001 MI *vs* sCAD; no significant differences were found between sCAD and HD). **Panel D:** Quantitative analysis of PKCε mRNA expression by real-time PCR. Equal amounts of total cDNA were amplified by PCR. The expression level of PKCε mRNA in each patient was compared with the mean expression in the HD group. Data from 10 patients of each group are shown (Mann Whitney test: p = 0.001 MI *vs* HD; p = 0.009 MI *vs* sCAD; no significant differences were found between sCAD and HD). **Panel E:** PKCε sequencing in MI patients. The presence of PKCε was further confirmed in MI patients by bi-directional sequencing. Representative fragment of the cDNA sequences (reverse strand) of PKCε encompassing the exons 1 and 2 (from nucleotide 11 to nucleotide 397). Patients #1, #3 and #7 belonging to the MI group showed the presence (in heterozygosity) of the rs12615152 (c.294C>T, p.Cys98Cys).

Since PKCε is finely modulated during megakaryocytopoiesis [34] and is known to increase the response of mouse platelets to GPVI-mediated activation [24], we reasoned that it could be a good candidate to account for the hyper-responsiveness of platelets from MI patients. Therefore, we extracted total mRNA from highly purified platelets and studied the expression of mRNA for PKCε by RT-PCR. The absence of nucleated cells contamination in the isolated platelet population was assessed by RT-PCR for CD45 expression (**Fig. 2A**). Moreover, given the dimensions (190 Kb) of the first intronic sequence of the PKCε gene and the positioning of the primers we use for PKCε RT-PCR, we could reasonably exclude the presence of PKCε pre-mRNA in platelets. In agreement with previous data [24], expression of PKCε mRNA was found at low frequency both in platelets from normal subjects and sCAD patients. On the contrary, platelets from the majority of MI patients (21 out of 24; 87.5%) had a clear-cut expression of PKCε mRNA (**Fig. 2B, C**), as confirmed also by qPCR (**Fig. 2D**). Retrotranscribed cDNA from platelet PKCε mRNA (MI patients) was bi-directionally sequenced in the region encompassing the exons 1 and 2 (*ENST00000306156*; nueclotides from 11 to 397). Analysis of the sequences and subsequent BLAST analysis revealed a truly concordance of cDNA with PKCε (**Fig. 2E**).

**Figure 3:**
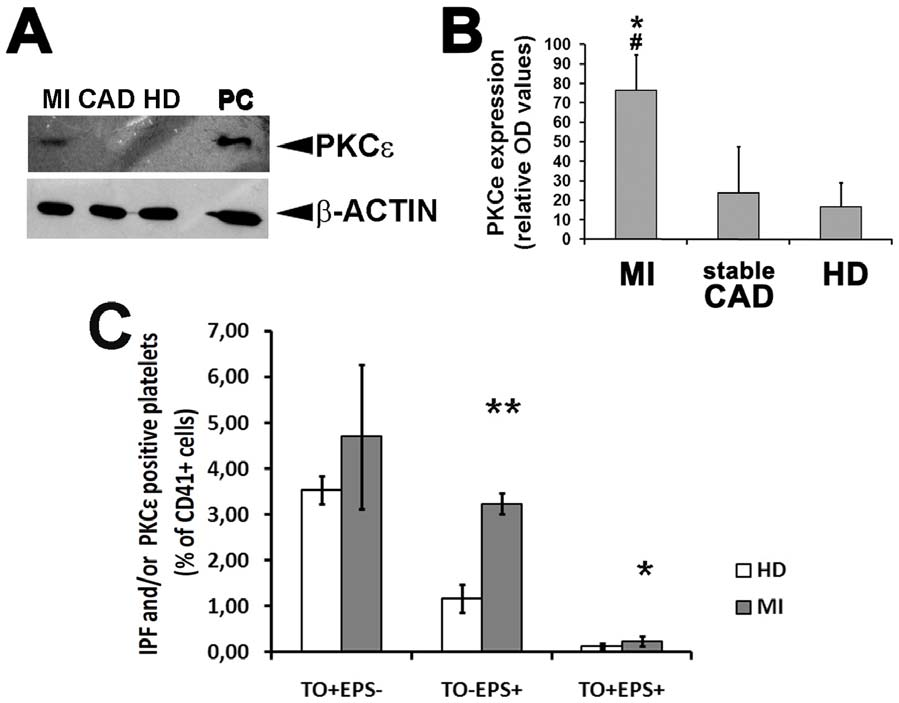
Platelets from MI patients express PKCε protein. **Panel A:** A representative Western blot assay for the detection of total PKCε protein expression in platelets from PKCε mRNA-positive MI, sCAD and HD. β-Actin was assayed for protein loading. **Panel B:** Densitometric analysis of PKCε protein expression normalized against β-actin in PKCε mRNA-positive MI, sCAD and HD platelets. Data are expressed as means ± S.D. (Anova and Bonferroni t-test; * P<0.05 MI *vs* HD; # P<0.05 MI *vs* sCAD; no significant differences were found between sCAD and HD). **Panel C:** PKCε protein expression in mature and immature platelets from healty donors (HD) and MI patients. Cells were simultaneously labelled with Thyazole Orange (TO) -to visualize the immature platelet fraction (IPF) - and anti-PKCε mAb, and analyzed by flow cytometry. Three populations were identified within the CD41+ cell subset: PKCε negative reticulated platelets (TO+EPS−); PKCε positive reticulated platelets (TO+EPS+); PKCε positive mature platelets (TO−EPS+); (Data from 3 patients/group, expressed as means ± S.D. t-Test ** P<0.001; * P<0.05).

We consequently studied the expression levels of PKCε protein by Western Blot selecting PKCε mRNA-positive patients from each group (10 patients from the MI group; 5 patients from the sCAD group; 3 subjects from HD group). As expected, accordingly with qPCR results, PKCε expression in MI patients was about three fold greater then sCAD and healty subjects (**Fig. 3A, B**).

To test if this “ectopic” expression of PKCε in the platelets of MI patients could be attributed to the immature platelet fraction, we analysed by flow cytometry the population of reticulated platelets from MI patients and HD. As reported in **Figure 3C**, the percentage of platelets from MI patients expressing PKCε protein was significantly increased both in mature (TO−EPS+) and immature (reticulated) (TO+EPS+) platelet fractions, as compared to HD. More specifically, the ratio between PKCε positive platelet subsets from MI *vs* HD subjects was 2.9±0.73 in mature platelets (TO−EPS+) and 2.17±1.44 in immature platelets (TO+EPS+) (p = 0.48, ns).

### Platelets from MI patients during follow up become negative for PKCε mRNA

To test whether the expression of PKCε in MI was transient or stable, 11 randomly selected MI patients positive for platelet PKCε mRNA expression were re-called between day 15 and day 30 from the acute episode and the platelets were tested again for PKCε expression. All platelet samples were found negative for PKCε mRNA expression as soon as 15 days from the acute MI episode (not shown).

### PKCε-expressing platelets are hyper-responsive

**Figure 4:**
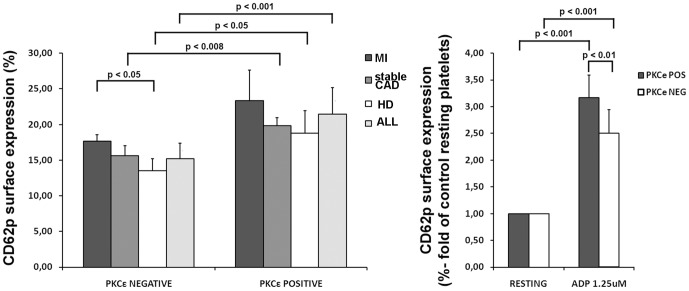
PKCε protein expression in platelets correlates with their activation levels. **Panel A:** Flow cytometric analysis of platelet CD62p surface expression in patients with MI, sCAD, healthy donor and in all the sample (ALL), on the basis of PKCε expression. Cells were stained with specific mAb anti P-selectin (CD62p). Seven patients were analyzed for each group (MI: 2 PKCε negative and 5 PKCε positive samples; sCAD: 4 PKCε negative and 3 PKCε positive samples; HD: 4 PKCε negative and 3 PKCε positive samples). Data is expressed as mean ± S.D (Anova and Bonferroni t-test). **Panel B:** Flow cytometric analysis of CD62p surface expression in PKCε negative and positive platelets. Cells were treated with ADP and compared with untreated platelets (resting). Ten patients were analyzed for each group. Data is expressed as mean ± S.D (Anova and Bonferroni t-test).

To study the correlation between PKCε expression and platelet activation, we compared the p-selectin (CD62p) cell surface expression in PKCε positive vs PKCε negative platelets in all the analysed groups (**Fig. 4A**). The general trend is a significant higher p-selectin expression in PKCε positive cells. Subsequently, we tested the platelet reactivity to the stimulation with sub-optimal doses of ADP. As expected, both PKCε negative and positive platelets were activated, as compared to controls (resting), but the positive cells were significantly more reactive than the negative (**Fig. 4B**)

### PKCε-overexpressing platelets are hyper-responsive and show enhanced adhesion to collagen

**Figure 5:**
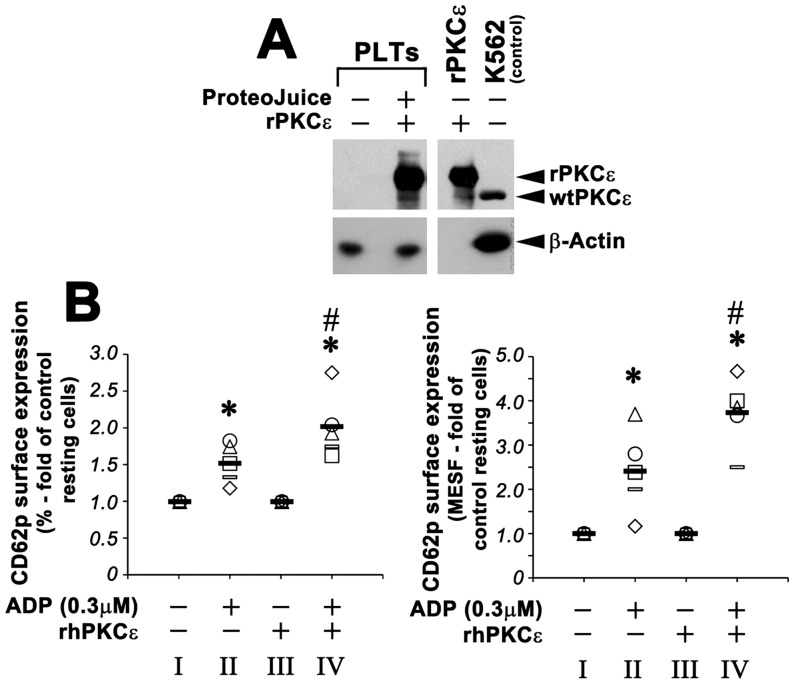
PKCε protein transfection in normal platelets induces hyper-responsiveness to ADP-mediated activation. **Panel A.** Western blot detection of total PKCε protein expression in transfected platelets. Healthy donor platelets were incubated with ProteoJuice medium in the presence or absence (negative control) of recombinant PKCε (rhPKCε). K562 cells were used as positive control. β-Actin was assayed for protein loading. **Panel B:** The expression of CD62p on the surface of activated platelets was compared to the expression of CD62p on resting platelets. rhPKCε-transfected platelets were significantly more reactive than activated control platelets. **Left panel** shows the percentage of positive cells. **Right panel** shows the absolute numbers of surface antigens expressed/cell (MESF). Data from 5 independent experiments (each symbol is related to one experiment) are expressed as means ± S.D. (Anova and Bonferroni t-test; * P<0.05 activated platelets *vs* resting platelets – II *vs* I and IV *vs* III; # P<0.05 rhPKCε-transfected platelets *vs* activated control platelets – IV *vs* II).

To study the functional role of PKCε in platelets, we subsequently decided to force its expression in platelets from normal healthy donors, in vitro mimicking the in vivo situation. rhPKCε protein was therefore successfully transfected in healthy donor platelets (thus originally negative for PKCε expression) (**Fig. 5A**). Subsequently, PKCε^+^ platelets were functionally assayed for ADP-induced activation and shear stress adhesion, using mock-transfected normal platelets as negative controls.

First, PKCε^+^ normal platelets and control platelets were treated with minimal doses of ADP (0.3 µM) and analyzed for CD62p surface expression by flow cytometry. There were no significant differences in the surface expression of CD62p between PKCε^+^ normal platelets and control platelets in the absence of ADP stimulation. On the contrary, PKCε^+^ platelets treated with 0.3 µM ADP express significantly higher levels of CD62p than control platelets (**Fig. 5B**).

**Figure 6:**
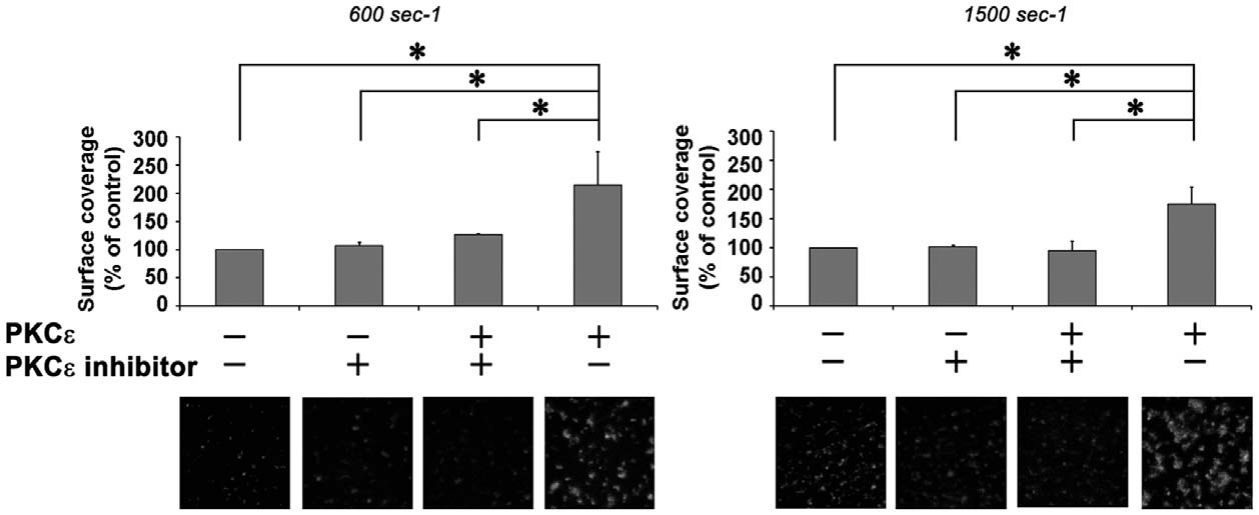
PKCε enhances platelet adhesion to fibrillar type I collagen under flow conditions. rhPKCε-transfected and control platelets were reconstituted in whole blood, previously deprived from PRP, and tested for their adhesion capacity under flow. Mepacrine-loaded platelets (5–7×10^8^/ml) and washed erythrocytes (hematocrit 42–45%) suspended in plasma, were perfused for 3 minutes over immobilized fibrillar type I collagen. Surface coverage was measured on an area of 0.07 mm^2^ after 3 minutes of perfusion at 600 s^−1^ or 1500 s^−1^ and is shown as mean ± 95% confidence intervals of at least 3 separate experiments. Results are shown relative to the values observed in untreated blood cell suspensions (control) (Anova and Bonferroni t-test; * p<0.01). Representative single-frame images of each surface are also shown.

Second, we studied the adhesion to collagen-coated surfaces of PKCε-overexpressing normal platelets under controlled physiological low (600 sec^−1^) or high (1500 sec^−1^) shear rates. Adhesion to collagen at 1500 sec^−1^ was significantly increased in normal platelets over-expressing PKCε, particularly after 3 minutes testing, (**Fig. 6**). Although hampered by a high variability, adhesion at low shear rate also showed a trend to increase. Adhesion of PKCε-transfected platelets treated with the PKCε inhibitor was similar to that of untreated platelets, demonstrating the specificity of the observed enhacement.

## Discussion

Platelets play a central role in the genesis and propagation of atherothrombosis and are therefore the target of several therapies for the prevention of thrombosis, particularly in the coronary artery district, where thrombi are the main responsible for myocardial infarction. Platelets are produced by megakaryocytes as anucleated cells that however retain the protein synthesis machinery, some small pre-mRNAs and selective mRNAs that can therefore be efficiently translated during the platelet life, that lasts around 10 days.

In general, PKC is established as an important regulator of several platelet functions. Of the many known PKC isoforms, not all are physiologically expressed in mature platelets, with some important differences between human and mouse platelets. It is now possible however to associate specific platelet functions to specific PKC isoforms. For instance, Konopatskaya et al. [48] recently demonstrated, using a genetic approach, the key role played by PKCα in α-granules release and thrombus formation, with no role in platelet adhesion to collagen. On the contrary, absence of PKCδ and PKCθ enhances the activation response to GPVI agonists and adhesion to collagen [46]. Moreover, the absence of PKCβ and θ abrogate outside-in signaling via αIIbβ3 integrin [11], [23]. Indeed, several receptors involved in platelet activation signal *via* PKC family members, like – for instance – thrombin or the collagen receptor GPVI. As a consequence, pharmacological inhibitors of PKC inhibit platelet aggregation.

In our hands, the majority of HD did not express PKCε in their platelets. This is in agreement with our previous data [34] describing the down-modulation of PKCε expression in *in vitro* human megakaryocytopoiesis from day 6 onward of TPO-driven MK differentiation of CD34 precursors. It is interesting however that mouse platelets – on the contrary – express PKCε at high levels. From the studies in mice we learn that PKCε plays a relevant role in the activatory signaling cascade emanating from the GPVI receptor. In fact, Pears et al. [24] elegantly demonstrated a marked reduction in the onset of aggregation and level of ATP secretion in response to collagen in the PKCε null mouse in the absence of collagen receptors alterations. In particular, the same Authors identify a role for PKCε in the GPVI signaling pathway, mediated by a reduction of FcRγ-chain phosphorylation.

The development of a platelet thrombus in coronary arteries is usually a critical, final phase in atherothrombosis, leading to MI. At the high shear rates typical of small arteries, the initial tethering of platelets at the sites of vascular injury or plaque rupture is mediated by GPIb/V/IX and collagen-immobilized VWF. This interaction – however – has a high off rate, and is not sufficient for stable adhesion and platelet activation, that are the key events for the formation of the trombus. The shift to stable adhesion requires the subsequent interaction between the platelet and the extracellular matrix proteins, notably between GPVI and collagen [49]–[51]. GPVI is a signal-transducing, non-integrin collagen receptor that mediates platelet activation, secretion of pro-coagulative factors and surface phosphatidylserine expression that promotes thrombin formation. Interestingly, Bigalke et al. recently demonstrated that platelet surface GPVI expression is already elevated hours before the onset of MI [50]. Genome wide association studies analyzed the *GPVI* gene in detail, finding large differences between populations and a relatively high number of sequence haplotypes which might account for the substantial inter-individual variation in the platelet response to collagen (or to Collagen Related Protein, CRP). Subsequent proteomic studies performed in high, mid and low responders showed something like 1,000 proteins, clustered in patterns, which included signaling as well as trafficking and transmembrane proteins [52]. We show that human platelet PKCε is selectively *de novo* expressed in MI, but not in sCAD patients, during the acute event while its expression returns negative after 15 days of follow-up. Functionally, we demonstrate that PKCε-transfected normal human platelets enhance their adhesion properties to collagen-coated surfaces under physiologically high shear forces. MI patients express PKCε mRNA at significantly higher frequency than HD and sCAD. Considering the dimensions of the first intronic sequence of the PKCε gene, that would virtually preclude the persistence of a potential PKCε pre-mRNA in the platelet, our findings suggest that platelet generations produced before the acute event of MI might retain PKCε-mRNA that is not down-regulated during terminal MK differentiation. An alternative explanation would be an anticipated release of platelets, before physiological PKCε down-modulation. This possibility is however unlikely, as PKCε down-modulation takes place around day 6 of *in vitro* MK differentiation, that would be too early. Besides, the analysis conducted on the reticulated platelets of some MI patients of our cohort did not show any difference in terms of of PKCε expression as compared to mature platelets, excluding the possibility that the appearance of PKCε positive platelets in MI patients could be selectively ascribed to newly formed platelets.

As a novel PKC isoform, PKCε would then boost platelet activation responding to DAG generation down-stream the immunoreceptor GPIV-FcRγ complex, that signals *via* cytoplasmic PLCγ, or any other activating surface receptor that signals *via* phosphoinositides breakdown. To this respect, one could speculate that an ectopic expression of PKCε accelerates platelet activation and perhaps the inside-out signaling that is essential to form thrombi. From a different perspective, the ectopic expression of PKCε in platelets could be used as a marker of probability to anticipate the acute event in patients at risk.
